# 2533: Beyond “REACH”: The Research, Education, And Community Health (REACH) coalition as an exemplar for broad-based stakeholder engagement

**DOI:** 10.1017/cts.2017.192

**Published:** 2018-05-10

**Authors:** Sharon A. Croisant, Christine Arcari, John Prochaska, Amber Anthony, Brittany Wallace, Chantele Singleton, Lori Wiseman, Rob Ruffner, Tino Gonzalez, Dwayne Jones, Fredia Marie Brown, Julie Purser, Allan Brasier

**Affiliations:** UTMB, Galveston, TX, USA

## Abstract

OBJECTIVES/SPECIFIC AIMS: The Institute for Transnational Sciences (ITS) has developed novel methods to ethically engage stakeholders across the transnational research spectrum, up to and including public health practice and policy. METHODS/STUDY POPULATION: In 2014, the ITS co-founded The Research, Education, And Community Health (REACH), the mission of which was to facilitate communication, collaborative research, and service activities between faculty and scientists and area community leaders. The intent was to identify and meet the needs of our communities without gaps and/or redundancies, thus better leveraging time, funding, and efforts. RESULTS/ANTICIPATED RESULTS: REACH now boasts 23 Centers, Departments, and Institutes, as well as 39 community organizations, including public and mental health agencies, clinicians, policy makers, family service centers, cultural and faith-based organizations, business, and local schools/colleges. We offer 3 methods for consideration as best practices: (1) a comprehensive community health needs assessment, (2) an “Offer and Ask” community/campus partnership mechanism, and (3) Community Science Workshops, based on the European Union’s Science Shops. DISCUSSION/SIGNIFICANCE OF IMPACT: Results of REACH’s work have been used to provide guidance for enhanced, data-driven programs and allocation of resources for local and statewide initiatives. The organization has evolved into an independent coalition seeking 501(c)3 status and is planning to expand its scope to 5 counties. REACH thus serves as model for successful replication across applicable CTSA hubs.

